# IVIVC for Extended Release Hydrophilic Matrix Tablets in Consideration of Biorelevant Mechanical Stress

**DOI:** 10.1007/s11095-020-02940-7

**Published:** 2020-10-22

**Authors:** Valentyn Mohylyuk, Seyedreza Goldoozian, Gavin P. Andrews, Andriy Dashevskiy

**Affiliations:** 1 Pharmaceutical Engineering Group, School of Pharmacy, Queen’s University Belfast, 97 Lisburn Road, Belfast, BT9 7BL UK; 2 College of Pharmacy, Freie Universität Berlin, Kelchstrasse 31, 12169 Berlin, Germany

**Keywords:** extended release, gastrointestinal contraction, hydrophilic matrix tablet, IVIVC, pharmacokinetic profile

## Abstract

**Purpose:**

When establishing IVIVC, a special problem arises by interpretation of averaged *in vivo* profiles insight of considerable individual variations in term of time and number of mechanical stress events in GI-tract. The objective of the study was to investigate and forecast the effect of mechanical stress on *in vivo* behavior in human of hydrophilic matrix tablets.

**Methods:**

Dissolution profiles for the marketed products were obtained at different conditions (stirring speed, single- or repeatable mechanical stress applied) and convoluted into C-t profiles. Vice versa, published *in vivo* C-t profiles of the products were deconvoluted into absorption profiles and compared with dissolution profiles by similarity factor.

**Results:**

Investigated hydrophilic matrix tablets varied in term of their resistance against hydrodynamic stress or single stress during the dissolution. Different scenarios, including repeatable mechanical stress, were investigated on mostly prone Seroquel® XR 50 mg. None of the particular scenarios fits to the published *in vivo* C-t profile of Seroquel® XR 50 mg representing, however, the average of individual profiles related to scenarios differing by number, frequency and time of contraction stress. When different scenarios were combined in different proportions, the profiles became closer to the original *in vivo* profile including a burst between 4 and 5 h, probably, due to stress-events in GI-tract.

**Conclusion:**

For establishing IVIVC of oral dosage forms susceptible mechanical stress, a comparison of the deconvoluted individual *in vivo* profiles with *in vitro* profiles of different dissolution scenarios can be recommended.

## Introduction

In addition to being used to understand oral bioavailability, *in vitro* - *in vivo* correlation (IVIVC) is being increasingly applied in the development of controlled release oral dosage forms. For example, an important and extensive cross-organization collaborative project involving multidisciplinary partners (OrBiTo) has examined important physicochemical properties of active pharmaceutical ingredients, *in vitro* characterization of drug formulations, characterization of *in vivo* behavior of compounds and formulations in GI-tract as well as in-silico models in developing oral dosage forms (1).

Recently, the effect of simulated GI-stress during dissolution from an extended release matrix tablet has been investigated using a specially adapted dissolution apparatus including different load cells (2–8), in-house dissolution testing apparatus (9–11), and periodical loading of dosage forms outside of the dissolution vessel (12,13). Commonly, a single mechanical stress event e.g. 300 mbar (3,6), 300 g/cm^2^ (13) or 400 g (10) during physiologically-relevant dissolution tests was applied in order to replicate as best as possible *in vivo* data obtained with SmartPill® (14). Using an alternative approach, a “destructive force dependent release system” (15), the maximum stomach and small intestine contraction force were utilized and determined at the level of approximately 1.9 N (16) and 1.2 N (17), respectively.

The transportation of tablet along the GI-tract is highly unpredictable with relatively high intra- and interindividual variability. Generally agreed periodicity of stomach emptying via migrating myoelectric is <120 min, however, it is not entirely predictable (18) due to, for example, a retro-propulsion back into the stomach of non-disintegrated dosage forms (19). The mechanical stress-events in the stomach and intestine can depend on many factors including individual physiological features, type of physical activity, lifestyle, and type of consumed food/drink (20). Also data regarding the effects of tablet size on stomach transit are conflicting (21,22). Thus, the behavior of a non-disintegrating tablet in GI-tract is unique in terms of residence time in different parts of GI-tract, the effect of different media, agitation level and number and severity of stress-events.

The importance of predictive dissolution testing is continuously increasing because of the possibility of pharmacokinetic modelling and deeper understanding of different factors affecting drug absorption *in vivo* (23). For this purpose, *in vivo* C-t profiles can be deconvoluted to an absorption profile and then compared to dissolution profiles (24). Because the dissolution is the rate-determining step for extended release dosage forms, a correlation of dissolution profile to absorption profile is justified (25). On another hand, dissolution profiles can be convoluted into C-t profiles and then compared with *in vivo* C-t profiles (24). One problem associated with both approaches is the relatively high spread on the individual *in vivo* data leading to misinterpretation based on averaged C-t profiles which represent the mean of different scenarios predetermined by level, number and frequency of the stress which varies in different parts of the GI-tract (14–17). Therefore, the comparison of either single or averaged *in vivo* with *in vitro* profiles would be preferable (26). Unfortunately, in most cases, individual C-t profiles are available only for sponsors of bioequivalence trials at fasting conditions, and generally available data often presents only averaged (geometric mean) profiles. Only a limited number of available sources allow an insight into original single C-t profiles and their variability e.g. a bioequivalence study with Seroquel® XR 300 mg (27).

Thus, it appears difficult to fit a specific dissolution scenario to averaged data. More logically it would be rational to generate dissolution profiles under different dissolution conditions, which mimic reasonable individual GI scenarios, and then convert them into predicted individual C-t profiles. Averaged predicted C-t profiles can be achieved by balancing of individual profiles in proportions reflecting the probability of the corresponding scenarios *in vivo*.

Since swellable/erodible hydrophilic tablets are considered as highly vulnerable to the mechanical stress, the objective of this study was to assess the effect of mechanical stress events in different dissolution scenarios and the impact that may have upon the prediction of *in vivo* profiles for different hydrophilic matrix tablets.

## Materials and Methods

### Materials

Commercial products Tromphyllin® retard 300 mg (Trommsdorff GmbH & Co. KG, Alsdorf, Germany), Glucophage® XR 500 mg (Merck Serono GmbH, Darmstadt, Germany), Alfuzosin-ratiopharm® uno 10 mg (Ratiopharm GmbH, Ulm, Germany), Seroquel® XR 50 mg (AstraZeneca GmbH, Wedel, Germany) and Preductal® MR 35 mg (Les Laboratories Servier, Gidy, France) were used. Chemicals used for preparation of dissolution media were of Pharmacopoeia grade and used as received.

### Dissolution

Since investigated extended-release hydrophilic matrix tablets supposed to release most of the active substance in the intestinal environment, USP phosphate buffer solution pH of 6.8 was used as the main dissolution medium. Dissolution testing was conducted using an USP Apparatus II (28) with a paddle agitation speed of 50, 100 or 150 rpm (VK 7000, VanKel Industries, NJ, USA) in 900 mL dissolution medium (29). If specified, at predetermined time-points (as after 1, 2 and 4 h) tablets were withdrawn together with 6 mL of medium, placed in Petri dishes and subjected to 2 N mechanical loading for 60 s using a texture analyzer (TA.XTplus, Stable Micro Systems Ltd., UK) equipped with a flat-faced cylindrical probe with a diameter of 20 mm. After that, tablets were immediately placed back in the dissolution vessel and residuals in the Petri dishes were rinsed with 5 mL of medium to continue the dissolution test. During the dissolution test, samples were taken at predetermined time-points: for Tromphyllin® retard 300, Glucophage® XR 500 mg – every 30 min during the first 6 h and then every hour; Alfuzosin-ratiopharm® uno 10 mg – every 30 min during the first hour and then every hour; Seroquel® XR 50 mg – at 15, 30, 60, 90, 120 min and then every hour; and Preductal® MR 35 mg – every 30 min during the first 7 h and then every hour. The schedule of mechanical stress loading was the same, but the sampling plan was for every commercial product individual because of the different duration and T_max_. Samples were filtered through a 0.22 μm filters and active substances were quantified using UV-spectroscopy (HP 8453, Agilent Technologies GmbH, Germany) with following parameters: theophylline - λ = 271 nm, C = 17.62*A, r^2^ = 0.9993; metformin λ = 234 nm, C = 14.64*A, r^2^ = 0.9997, alfuzosin - λ = 245 nm, C = 9.11*A + 0.0447, r^2^ = 0.9998, trimetazidine dihydrochloride - λ = 269 nm, C = 45.46*A, r^2^ = 0.9999, quetiapine fumarate λ = 290 nm, C = 58.14*A, r^2^ = 0.9999. Drug release (%), presented as a mean of six parallel measurements, was plotted *versus* time.

### Deconvolution and Convolution

Published original single dose C-t profile of Glucophage® XR 500 mg (30), Alfuzosin-ratiopharm® uno 10 mg (31), Tromphyllin® retard 300 mg (31) Preductal® MR 35 mg (32) and Seroquel® XR 50 mg (31) at fasting conditions have been shown to follow a one compartmental distribution model and, therefore, deconvoluted using the Wagner-Nelson equation (Eq. 1) (33, 34) and experimental dissolution profiles were convoluted into C-t profiles using the following equation (Eq. 2) (33, 34)

1 $$ F\ \left(\%\right)=\frac{C_p+{k}_{el}{AUC}_{0-t}}{k_{el}\ {AUC}_{\mathit{\operatorname{inf}}.}} $$

2 $$ {C}_{p+1}=\frac{\frac{2\cdotp \Delta  F\cdotp D}{V_d}+{C}_p\cdotp \left(2-{k}_{el}\cdotp \Delta  t\right)}{\left(2-{k}_{el}\cdotp \Delta  t\right)} $$

where: F is cumulative fraction of drug absorbed at time t; C_p_ is the plasma concentration of drug at time t; *k*_*el*_ = *ln* 2/*t*_1/2_ is the elimination rate constant; AUC_0- t_ is the area under the plasma concentration-time (C-t) profile until time t; D is a dose corrected on bioavailability; and V_d_ is apparent volume distribution.

### Profile Comparison

For comparison of profiles the similarity factor (f2) was calculated using the following equation:

3 $$ f2=50\cdotp \mathrm{Log}\left({\left[1+\frac{1}{n}\sum {\left({C}_{diss.}-{C}_{abs.}\right)}^2\right]}^{-0.5}\times 100\right) $$

where *C*_*diss*_ represents data from dissolution profiles achieved experimentally at different conditions and *C*_*abs.*_ – data from absorption profiles achieved by deconvolution of published original profiles.

Similarity factors, for each investigated product, achieved for dissolution profiles at a different stirring speed *vs*. absorption profile were compared with each other by calculation of a difference between the maximal f2_max_ value in the set and f2_i_ of interest. The extent and direction of change in dissolution profiles upon single stress were expressed by a difference between f2_stress_ and f2_no stress_. Alternatively, linear regression analysis was applied to the *in vitro*–*in vivo* correlation plots and the coefficient of determination (r^2^) was calculated using the following equation:

4 $$ {r}^2={\left(\frac{n\sum \left({x}_i{y}_i\right)-\sum {x}_i\sum {y}_i}{\sqrt{\left[n\sum \left({x}_i^2\right)-{\left(\sum {x}_i\right)}^2\right]\cdotp \left[n\sum \left({y_i}^2\right)-{\left(\sum {y}_i\right)}^2\right]}\ }\right)}^2 $$

where: *x*_*i*_ – fraction dissolved and *y*_*i*_ fraction absorbed at respective time points.

## Results

For comparison of release and deconvoluted curves, the correlation coefficient (r2), the similarity factor (f2), the mean dissolution time (MDT) or, for comparison of *in vivo* and convoluted release curves, the AUC are usually used. MDT is an integral parameter hiding essential information about shape of dissolution profile. A precise calculation of AUC in this study was, unfortunately, not possible because the *in vivo* data available (only partially) in the literature were used. Therefore, the correlation coefficient and similarity factor were applied for comparison of the dissolution and deconvoluted *in vivo* profiles.

### Effect of Hydrodynamic Conditions

**Fig. 1:**
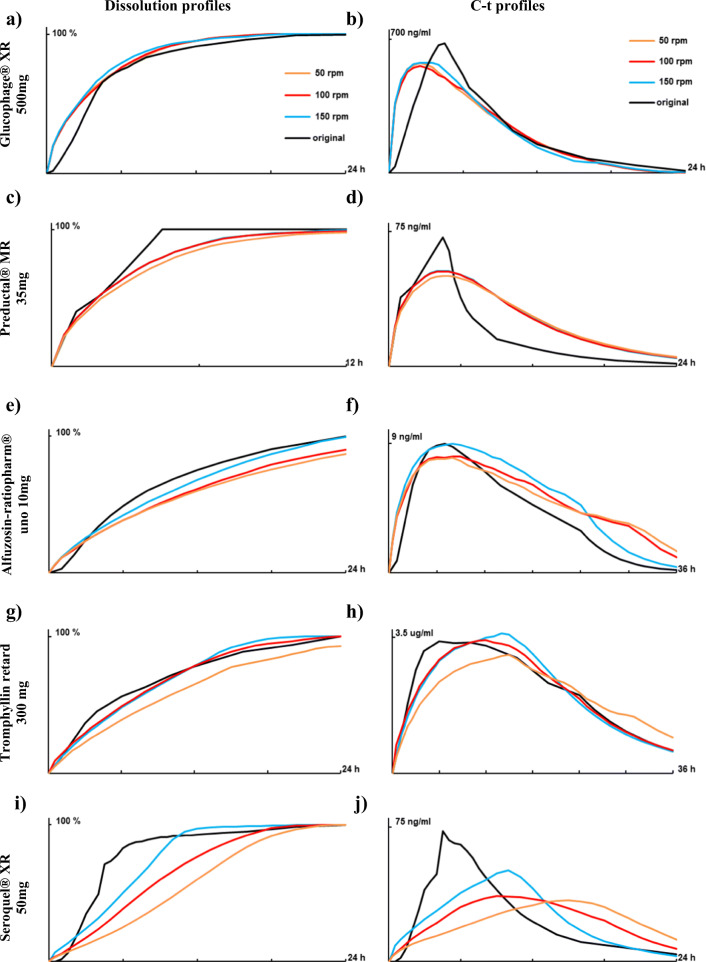
(**a**, **c**, **e**, **g** and **i**) Dissolution profiles at stirring speed in phosphate buffer pH 6.8 in comparison with deconvoluted original C-t profiles. (**b**, **d**, **f**, **h** and **j**) convoluted profiles at respective dissolution condition in comparison with original C-t profiles. (**a** and **b**) Glucophage® XR 500 mg, (**c** and **d**) Preductal® MR 35 mg, (**e** and **f**) Alfuzosin-ratiopharm® uno 10 mg, (**g** and **h**) Tromphyllin® retard 300 mg and (**i** and **j**) Seroquel® XR 50 mg. SD has been waived for clarity of graphs.

To investigate the effect of paddle stirring speed, the f2 value for dissolution profiles at stirring speed 50, 100 and 150 rpm *vs*. corresponding absorption profile (deconvoluted published profiles) were calculated. Drug release from Glucophage® XR and Preductal® MR was not affected by paddle stirring speed in the range 50–150 rpm (Fig. 1a and c). The f2 was in a range 46.6–49.3 and 50.6–57.4 for Glucophage® XR and Preductal® MR, respectively (Table I). For the discrimination of existing differences between particular dissolution profiles, the f2 factor seems to be more powerful than r^2^ – coefficient of determination (Table I), which is, however, mostly used for quantification of IVIVC (35). A moderate effect of paddle stirring speed on drug release was observed for Alfuzosin-ratiopharm® and Tromphyllin® retard (Fig. 1e and g). For Alfuzosin-ratiopharm®, the f2 increased (47.1, 49.2 and 59.2) with increasing the stirring speed (50, 100 and 150 rpm), respectively (Table I). For Tromphyllin®, the dissolution profile at 100 rpm showed the greatest similarity with absorption profile (f2 = 65.7), followed by dissolution profile at 150 rpm (f2 = 61.0) and 50 rpm (f2 = 44.3) (Fig. 1g, Table I).

**Table I:** Similarity Factor (%) Comparing Absorption Profiles with Dissolution Profiles at Different Stirring Speed or Mechanical Stress

Products	Stirring speed				Mechanical stress			
	rpm	f2	f2_max_ - f2_i_^*^	r^2^	Stress after	f2	f2_stress_ - f2_no stress_^**^	r^2^
Glucophage XR 500 mg	50	48.3	1.0	0.990	1 h	47.3	−2.0	0.993
	100	49.3	0.0	0.990	2 h	49.2	−0.1	0.993
	150	46.6	2.7	0.995	4 h	47.7	−1.6	0.993
Preductal MR 35 mg	50	50.6	6.8	0.988	1 h	63.4	6.2	0.975
	100	57.2	0.2	0.987	2 h	67.9	10.7	0.984
	150	57.4	0.0	0.987	4 h	56.4	−0.8	0.991
Alfuzosin-ratiopharm uno 10 mg	50	47.1	12.1	0.989	1 h	52.5	3.3	0.989
	100	49.2	10.0	0.987	2 h	55.9	6.7	0.991
	150	59.2	0.0	0.987	4 h	59.5	10.3	0.990
Tromphyllin retard 300 mg	50	44.3	21.4	0.981	1 h	56.3	−9.4	0.989
	100	65.7	0.0	0.992	2 h	59.3	−6.4	0.990
	150	61.0	4.7	0.990	4 h	59.6	−6.1	0.990
Seroquel® XR 50 mg	50	21.4	13.7	0.581	1 h	34.6	6.9	0.864
	100	27.7	7.4	0.759	2 h	36.8	9.1	0.893
	150	35.1	0.0	0.853	4 h	43.9	16.2	0.925

*f2_max_- f2_i_ – the difference between maximal f2 value in the set and f2 of interest,

**f2_stress_ - f2_no stress_ – the difference between f2 for dissolution at 100 rpm with particular stress and without stress, respectively

Dissolution profiles of Seroquel® XR demonstrated the highest discrepancy in release at different paddle stirring speed (Fig. 1i). The f2 for comparison of dissolution/absorption profiles was rather low but increased (21.4, 27.7 and 35.1) with increasing the stirring speed (50, 100 and 150 rpm), respectively (Table I). Also, the shape of dissolution and absorption profiles differed considerably. Namely, dissolution profiles were continuous, while a pronounced lag time followed by steep rise was observed on absorption profile (Fig. 1i) which was derived from a sharp peak at 4.5 h on original *in vivo* C-t profile (Fig. 1j).

### Effect of Single Mechanical Stress Event

**Fig. 2:**
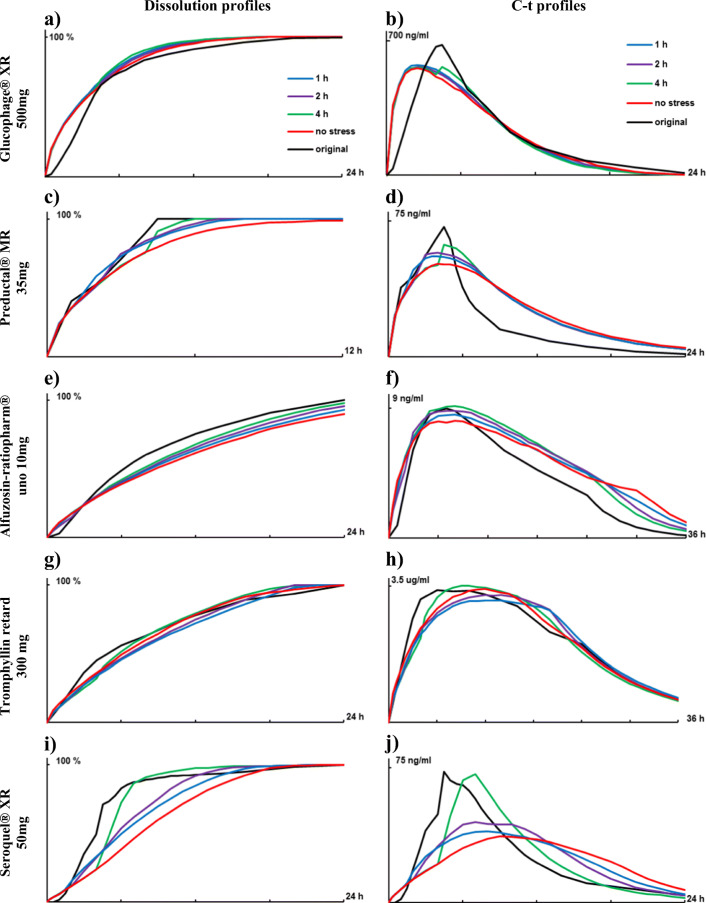
(**a**, **c**, **e**, **g** and **i**) Dissolution profiles at 100 rpm in phosphate buffer pH 6.8 and applied stress after 1, 2 or 4 h in comparison with deconvoluted original C-t profiles. (**b**, **d**, **f**, **h** and **j**) convoluted profiles at respective dissolution condition in comparison with the original C-t profile. (**a** and **b**) Glucophage® XR 500 mg, (**c** and **d**) Preductal® MR 35 mg, E and F) Alfuzosin-ratiopharm® uno 10 mg, (**g** and **h**) Tromphyllin® retard 300 mg and (**i** and **j**) Seroquel® XR 50 mg. SD has been waived for clarity of graphs.

To investigate the effect of a single mechanical stress event on dissolution, tablets were subjected to 2 N mechanical loading for 60 s after 1, 2 or 4 h. Under these conditions, dissolution profiles of Glucophage® XR, Alfuzosin-ratiopharm® and Tromphyllin® retard demonstrated relatively low changes (Fig. 2). Accordingly, convoluted C-t profiles had similar trends, although did not match the original profiles (Fig. 2). Consequently, f2 values for profiles with single stress events after 1, 2 and 4 h *vs*. non-stressed profiles changed only slightly (Table I).

For Preductal® MR and Seroquel® XR, each individual stress event caused a burst release. The most pronounced burst release was after mechanical stress at 4 h (Fig. 2c and i). Thus, for Preductal® MR, convoluted C-t profile matched well the original in term of t_max_ (approx. 4.5 h) unlike Cmax which was below of original (Fig. 2d). The highest burst release after 4 h was also in case of Seroquel® XR. The convoluted C-t profile with applied stress at 4 h was closest to original in term of Cmax but 1.5–2.5 h delayed (Fig. 2j).

### Effect of Repeatable Mechanical Stress Events

**Fig. 3:**
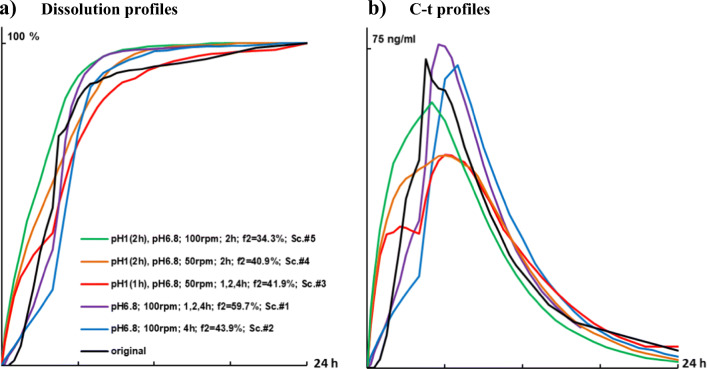
(**a**) Dissolution profiles upon consequent stress application after 1, 2 and 4 h during dissolution in 0.1 N HCl and/or phosphate buffer solution pH 6.8 and stirring speed 50 or 100 rpm in comparison with deconvoluted original C-t profile of Seroquel® XR 50 mg. (**b**) convoluted profiles at respective dissolution condition in comparison with the original C-t profile. SD has been waived for clarity of graphs.

Dissolution tests with repeatable mechanical stress events were performed with Seroquel® XR matrix tablets since they were most affected by mechanical stress. The highest f2 value (59.7%) was obtained using phosphate buffer solution pH 6.8 at 100 rpm and consequent stress application after 1, 2 and 4 h (Fig. 3a). Due to application of repeatable stress in the time range 1–4 h, C-t profiles became closer in the shape to the original (Fig. 3).

### Combination of Convoluted C-T Profiles

**Fig. 4:**
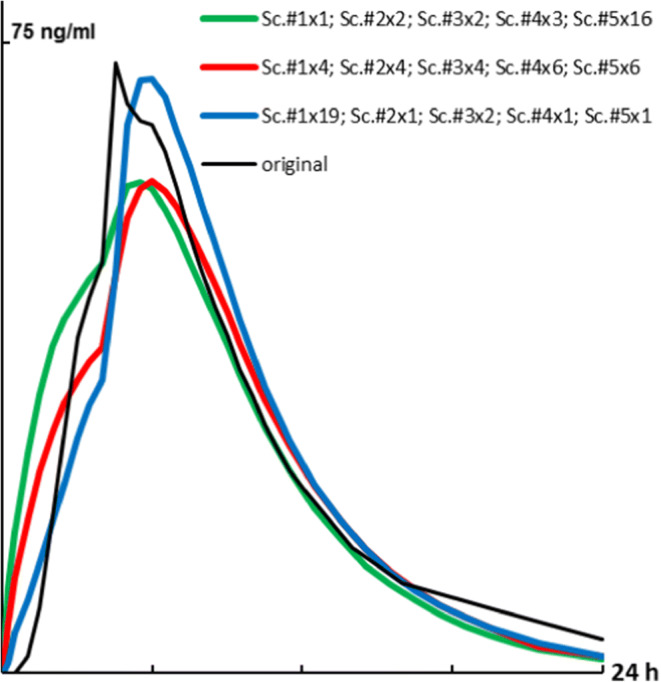
Predicted C-t profiles (weighted geometrical average of different release scenarios - Table III) in comparison with the original C-t profile of Seroquel® XR 50 mg.

In order to mimic data aggregation within one bioequivalence study, profiles according to 5 different *in vitro* dissolution scenarios (Fig. 4, Table II) were convoluted and combined in different ratios to create weighted averaged C-t profile. Generally, the shape of these profiles was closer to the original profile except for the difference in a lag-time (Fig. 4). The lag-time of the original profiles can be attributed to a gradual transport of gastric liquid with dissolved drug towards the small intestine before absorption. This transport was not considered by direct convolution.

**Table II:** Summary of Dissolution Profiles of Seroquel® XR 50 mg at Different Dissolution Conditions

Dissolution conditions			f2, %	Scenario
Medium pH	Stirring speed, rpm	Stress application after		
pH 6.8	100	1, 2 and 4 h	59.7	Sc. #1
pH 6.8	100	4 h	43.9	Sc. #2
pH 1 (1 h), pH 6.8	50	1, 2 and 4 h	41.9	Sc. #3
pH 1 (2 h), pH 6.8	50	2 h	40.9	Sc. #4
pH 6.8	100	2 h	36.8	n.a.
pH 6.8	150	no stress	35.8	n.a.
pH 6.8	100	1 h	34.6	n.a.
pH 1 (2 h), pH 6.8	100	2 h	34.3	Sc. #5
pH 1.0	100	1, 2 and 4 h	29.5	n.a.
pH 6.8	100	no stress	27.7	n.a.

n.a. not applied

Similarity factor (f2) compares the dissolution profile under respective conditions and deconvoluted original profile of Seroquel® XR 50 mg

## Discussion

All chosen commercial products differ in type and concentration of hypromellose, as a matrix-forming agent, weight, and shape of the tablets (36). One of the most common methods to investigate the robustness of swellable/erodible matrix tablets towards mechanical stress in the GI-tract can be dissolution testing under increased stirring speed (37). Generally, the best similarity of absorption and dissolution profiles was at higher stirring speed e.g. 100 or 150 rpm (Table I). General pharmacopeial dissolution test for tablets with USP Apparatus II recommends stirring speed 50 rpm, probably, not sufficiently mimicking the *in vivo* hydrodynamics which is especially critical for hydrophilic matrix tablets.

Further source of mechanical stress on dosage forms in the GI-tract are periodic contractions in stomach and intestine with a force of approximately 1.9 N (16) and 1.2 N (17), respectively. The mechanical stress was applied at 1, 2 and 4 h to cover time range when tablets can be affected by stomach/pyloric or upper intestinal contractions at fasting conditions. For some investigated products, namely, Glucophage® XR, Alfuzosin-ratiopharm® uno and Tromphyllin® retard dissolutions profiles were almost unchanged when subjected to 2 N mechanical loading after 1, 2 or 4 h during dissolution test. This robustness was, probably, the reason for relatively low T_max_ variability *in vivo* (Table III).

**Table III:** T_max_ Variability Sourced from Single-Dose Bioequivalence Trails at Fasten Conditions

API	Product	Subjects number	T_max_ (h) ± SD (range)	Ref.
Metformin hydrochloride	Glucophage® XR, 500 mg	18	4.4 ± 0.7	(30)
	Glucophage® XR, 750 mg	78	3.8 ± 1.2	(38)
Trimetazidine dihydrochloride	Preductal® MR, 35 mg	8	3 ± 1.5	(32)
		24	3.2 ± 1.3	(39)
Quetiapine fumarate	Seroquel® XR, 300 mg	24	5.0 (2.5–10)	(27)
		24	5.0 (0.9–20)	(40)
	Seroquel® XR, 200 mg	18	5.6 ± 2.0	(41)
	Seroquel® XR, 50 mg	≥12	6.0	(31)

Vice versa, for Seroquel® XR and Preductal® MR, the effect of mechanical stress was considerable being more pronounced when applied late (e.g. 4 h). This is because hydrophilic matrix tablets become softer upon swelling over time. Different robustness of investigated products can be explained by their formulations. Robust formulations contain hypromellose of higher molecular weight and content more than 20% *w/w* which allows the formation of gel with sufficient gel strength at the gel-solution interface over a long time period.

Trying to mimic biorelevant conditions, repeatable mechanic stress events need to be considered. This was investigated on the product most susceptible to mechanical stress in this study, namely, Seroquel® XR. Because of the markable pH-dependent solubility of quetiapine fumarate, the dissolution of Seroquel® XR was performed, optionally, in 0.1 N hydrochloric acid solution in first hour before changed to phosphate buffer solution pH 6.8. Due to application of repeatable stress in the time range 1–4 h, C-t profiles became closer in the shape to the original (Fig. 3) which was, most likely, predetermined repeatable stomach and intestinal stress. Unfortunately, none of the individual dissolution scenarios described satisfactorily the original profile of Seroquel® XR 50 mg tablets. Therefore, convoluted dissolution profiles (scenarios) were combined in different proportions, averaged, and compared with the original C-t profile (Fig. 4, Table II). The shape of all these profiles was closer to the published original ones. Some shape similarities, including the burst between 4 and 5 h, probably, due to stress-events in GI-tract, were also observed in other bioequivalence trials with Seroquel® XR tablets - 50 mg (31,40,42), − 200 mg (43) and - 300 mg (41). However, a perfect matching of burst times barely possible because *in vivo* profiles are always representing the average of particular studies with a limited number of subjects and very high intra/interindividual variability. Insight into individual profiles for Seroquel® XR 300 mg reveals that the burst can occur at 2, 3, 4, 5, 6 or even 10 h (27). In general, the variations of T_max_ *in vivo* for products susceptible mechanical stress were more pronounced in comparison with stress-resistant e.g. Glucophage® XR (Table III). Thus, for establishing IVIVC of oral dosage forms susceptible to mechanical stress, a comparison of the deconvoluted individual *in vivo* profiles with individual *in vitro* profiles of different release scenarios would be preferable. Further, a set of *in vitro* scenarios in different proportions can be used to achieve averaged profiles.

## Conclusion

Different marketed products formulated as swellable/erodible matrix tablets were investigated in terms of robustness against biorelevant mechanical stress. As robust formulations, Glucophage® XR 500 mg, Alfuzosin-ratiopharm® uno 10 mg or Tromphyllin® retard 300 mg were identified. These formulations had relatively low interindividual variability and a correlation of *in vitro* profiles generated with conventional dissolution tests with averaged *in vivo* profiles can be established. For formulations susceptible to mechanical stress e.g. Seroquel® XR 50 mg, a pharmacopeial dissolution test at the paddle speeds 50 vs. 150 rpm would give roughly an insight into robustness of matrix tablets against gastrointestinal mechanical stress. The IVIVC would be more successful if deconvoluted individual *in vivo* profiles were compared with *in vitro* profiles generated with different scenarios of release including the mechanical stress.

## Abbreviations

GI: Gastrointestinal
C-t: Concentration-time
IVIVC: *In vitro - in vivo* correlation
